# Research on Network Security Situation Awareness Based on the LSTM-DT Model

**DOI:** 10.3390/s21144788

**Published:** 2021-07-13

**Authors:** Haofang Zhang, Chunying Kang, Yao Xiao

**Affiliations:** School of Data Science and Technology, Heilongjiang University, Harbin 150000, China; 2191833@s.hlju.edu.cn (H.Z.); 2202527@s.hlju.edu.cn (Y.X.)

**Keywords:** network security situation assessment, analytic hierarchy process, stack sparse auto-encoder, long short-term memory network, decision tree

## Abstract

To better understand the behavior of attackers and describe the network state, we construct an LSTM-DT model for network security situation awareness, which provides risk assessment indicators and quantitative methods. This paper introduces the concept of attack probability, making prediction results more consistent with the actual network situation. The model is focused on the problem of the time sequence of network security situation assessment by using the decision tree algorithm (DT) and long short-term memory(LSTM) network. The biggest innovation of this paper is to change the description of the network situation in the original dataset. The original label only has attack and normal. We put forward a new idea which regards attack as a possibility, obtaining the probability of each attack, and describing the network situation by combining the occurrence probability and attack impact. Firstly, we determine the network risk assessment indicators through the dataset feature distribution, and we give the network risk assessment index a corresponding weight based on the analytic hierarchy process (AHP). Then, the stack sparse auto-encoder (SSAE) is used to learn the characteristics of the original dataset. The attack probability can be predicted by the processed dataset by using the LSTM network. At the same time, the DT algorithm is applied to identify attack types. Finally, we draw the corresponding curve according to the network security situation value at each time. Experiments show that the accuracy of the network situation awareness method proposed in this paper can reach 95%, and the accuracy of attack recognition can reach 87%. Compared with the former research results, the effect is better in describing complex network environment problems.

## 1. Introduction

Although the high-speed interaction of information facilitates our work and life, it also brings various problems related to network security. On 26 September 2020, China’s national Internet Emergency Response Center (CNCERT) released an analysis report on China’s Internet network security monitoring data in the first half of 2020. The report points out that in the first half of 2020, the number of computer malware samples captured was about 18.15 million, and the number of hosts infected with computer malware in China was about 3.04 million, an increase of 25.7%.

Against this background, network security situation awareness (NSSA) technology arises at a historic moment. Endsley [1] put forward the concept of situation awareness for the first time in 1988, defining it as “recognizing and understanding environmental factors under certain time and space conditions, and predicting the development trend of future things”. He designed the three-layer perceptual model: situation element extraction (Level 1), situation understanding (Level 2), and situation prediction (Level 3). In 1999, Bass [2] first proposed the concept of network security situation awareness by combining intrusion monitoring with network attack and defense.

In recent years, combined with a variety of technical means, many scholars have proposed their own NSSA scheme. Zhong [3] proposes an intrusion detection model based on a random forest (RF)-based feature selection approach and neural networks model. Referring to the idea of constructing a hybrid model, we use the SSAE network to learn the characteristics of the dataset, and then use the LSTM network to obtain the probability of each attack and make it assist the DT algorithm in identifying attacks.

Zhong’s [3] method only identifies the types of attacks but cannot explain the impact of each attack. Qian et al. [4] aligned the process of NSSA with the life cycle of security data and analyzed its needs, proposing a multilevel analysis framework, and used the RF algorithm to construct an evaluation model. It evaluates the network security situation from the perspective of the security data life cycle, which makes the NSSA more objective and accurate than the former. This paper chooses to evaluate the network security situation from the perspective of attack and analyzes different types of attacks from two aspects, attack impact and attack probability, which makes the NSSA process more complete and the comprehensive comparison results better.

### 1.1. Main Contributions

To improve the accuracy and objectivity of NSSA, this paper proposes a new NSSA model, that is, the LSTM-DT model. The main contributions of this paper are summarized as follows:

- This paper proposes a new NSSA method, which perceives the network security state from three aspects: network situation factor extraction, network situation assessment, and network situation prediction.
- We propose the concept of “attack probability”, which changes the previous scholars’ limitation of situation awareness only being able to identify attack types, and makes the final situation value more accurate.
- We propose the concept of “the influence degree of each network attack” and evaluate it by various methods, which makes the identified attack types have a more objective expression.
- The model has good stability, and the prediction accuracy can reach 95% when describing the general network environment, while the accuracy can still remain above 80% when describing the complex network environment.

### 1.2. Structure of the Paper

The rest of the paper is organized as follows. First, we present the literature review in Section 2. Then, we introduce the related model and algorithms in Section 3. We describe the design of the LSTM-DT hybrid model in Section 4. Section 5 presents the extraction and evaluation of network security situation elements. Section 6 presents the network security situation prediction algorithm. We propose datasets and experiment methods in Section 7. In Section 8, we analyze and compare the NSSA method proposed in this paper. Finally, some conclusions are given in Section 9.

## 2. Related Works

In order to better protect the network security, more and more scholars are engaged in the research on the NSSA. Yu et al. [5] propose a network security architecture based on immunology. Zhu et al. [6] describe a NSSA method from the perspective of big data, which includes network security situation detection (NSSD), network security situation understanding (NSSU), and network security situation projection (NSSP).

Based on the three-layer perceptual model of situation awareness proposed by Endsley, we divide the NSSA into three parts: situation element extraction, situation assessment, and situation prediction. The extraction of network security situation elements refers to the extraction of relevant elements in the network according to the established evaluation indexes. Articles [7,8,9] provide network security situation elements from different aspects, but they all have their own limitations. Network security situation assessment refers to dealing with the assessment indicators according to certain rules and analyzing the security incidents. The classic network situation assessment methods include the AHP, fuzzy analytic hierarchy process (FAHP), and set pair analysis. Ji et al. [10] use a method about network security situation assessment based on FAHP. Zhi et al. [11] propose a network security level protection evaluation method based on fuzzy synthesis and AHP (determine the evaluation interval, realize quantitative standardization, objective level protection evaluation method).

Network security situation prediction means predicting the future network situation according to the current network state and historical data. In recent years, the rapid development of machine learning provides a new solution for network situation prediction, such as the support vector machine (SVM) [12] and hidden Markov model (HMM) [13]. Then, deep learning applies to network security situation assessment: for example, [14] summarizes the artificial intelligence related to network security as well as the progress and challenges of current research; [15] studies the performance of different neural networks in the NSSP; and [16] proposes an LSTM network security situation prediction model based on the sigmoid weighted reinforcement mechanism, which can improve the convergence rate.

## 3. Related Model and Algorithms

### 3.1. Stack Sparse Auto-Encoder

The auto-encoder (AE) is an effective nonlinear dimensionality reduction method proposed by Hinton [17]. It uses the nonlinear transformation of the hidden layer in the neural network to map the original high-dimensional features. The basic self-encoder only reconstructs the original data, and the output layer data are simply copied to the output layer. The sparse auto-encoder (SAE) adds a sparsity constraint on the activation of hidden layer neurons in the self-encoder model [18]. The encoder uses the average activation value of hidden layer neuron output to constrain, using KL divergence to make it close to a given sparse value, and adds it as a penalty term to the loss function [19]. The loss function of the sparse self-encoder is calculated as follows:

(1) $$\begin{array}{c} L_{\mathrm{sparse}\;}=L_{E}+\beta \sum_{j}\mathrm{KL}\left(\rho ∥\rho_{j}^{∼}\right)\\ =\frac{1}{2}\sum_{k=1}^{N}\sum_{i=1}^{c}|x_{i}^{k}-{\left.y_{i}^{k}\right|}^{2}\\ +\beta \sum_{j}\mathrm{KL}\left(\rho ∥\rho_{j}^{∼}\right) \end{array}$$

where N is the total number of input samples *x*, and *C* is the feature dimension of samples. The purpose of the self-encoder is to make the raw data $x^{k}$ and the reconstructed data $y^{k}$ more similar: $x_{i}^{k}$ and $y_{i}^{k}$ represent the j-th feature respectively. $L_{E}$ is usually the mean square error function, which represents the loss function of the basic self-encoder. In the loss function formula, ρ is the sparsity parameter, $\rho_{j}^{∼}$ is the average activation value of the j-th neuron, and β is the penalty factor. $\mathrm{KL}\left(\rho ∥\rho_{j}∼\right)$ represents the penalty term, representing the KL divergence between $\left(\rho \tilde{j},1-\rho \tilde{j}\right)$. The specific formula is as follows:

(2) $$\begin{array}{c} \rho_{j}^{∼}=\frac{1}{2}\sum_{k=1}^{N}\left[h_{j}\left(x^{k}\right)\right]\\ \mathrm{KL}\left(\rho ∥\rho_{j}^{∼}\right)=\rho \log\frac{\rho }{\rho_{j}}\\ +\left(1-\rho \right)\log\frac{1-\rho }{l-\rho \tilde{j}} \end{array}$$

**Figure 1:**
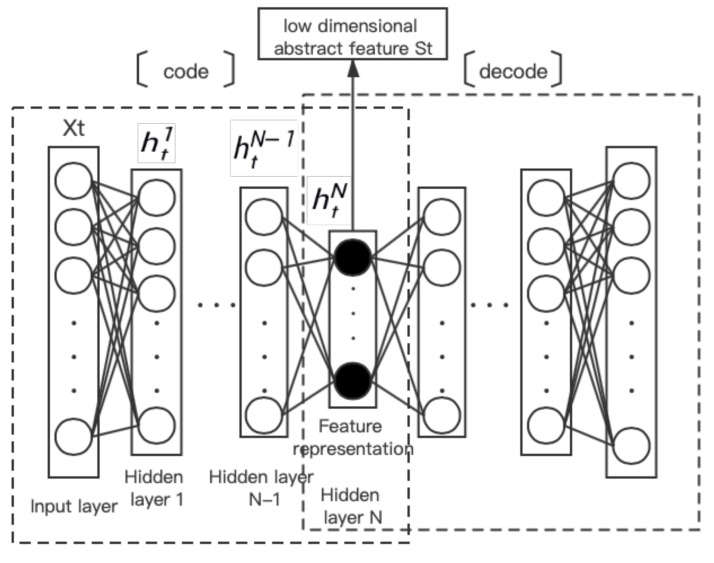
The network structure of SSAE.

The stack sparse auto-encoder is composed of multiple sparse self-encoders stacked and connected with different classifiers [20]. In this paper, the SSAE network unit is used to extract low-dimensional features from the data of each time in the extended sequence ($X_{t-m}$,…,$X_{t-2}$,$X_{t-1}$), and the feature sequence ($S_{t-m}$,…,$S_{t-2}$,$S_{t-1}$) is obtained, so as to achieve the purpose of feature learning. Its structure is shown in Figure 1.

The SSAE network inputs data $x_{t}$ into the first SAE to obtain the low-dimensional feature representation of hidden layer vector $h_{t}^{1}$, then inputs $h_{t}^{1}$ into the second SAE to obtain the low-dimensional feature representation of hidden layer vector $h_{t}^{2}$, and so on, obtaining the hidden layer vector $h_{t}^{N}$ as the last low-dimensional feature.

### 3.2. LSTM and DT

The LSTM network [21] is an improved recurrent neural network (RNN) which is used to process sequential signals. It adds memory units to each neural unit in the hidden layer based on the RNN, so that the memory information in the time series can be controlled. Several controllable gates (forgetting gate, input gate, and output gate) are used to transmit information among the units in the hidden layer, which can control the memory and forgetting degree of the information. The LSTM unit calculates the output value *h* of the hidden layer at time *t* according to the state value $c_{t-1}$ of the memory unit at time t−1, the output value $h_{t-1}$ of the hidden layer at time t−1, and the input value $x_{t}$ at time *t*. According to the flow direction of the signal, the specific calculation rules are as follows:

(3) $$\begin{array}{c} f_{t}=\sigma \left(W_{fx}x_{t}+W_{f_{t}}h_{t-1}+b_{f}\right)\\ i_{t}=\sigma \left(W_{ix}x_{t}+W_{ih}h_{t-1}+b_{i}\right)\\ g_{t}=\varphi \left(W_{gx}x_{t}+W_{gh}h_{t-1}+b_{g}\right)\\ c_{t}=c_{t-}⊙f_{t}+g_{t}⊙i_{t}\\ o_{t}=\sigma \left(W_{ox}x_{t}+W_{oh}h_{t-1}+b_{o}\right)\\ h_{t}=\varphi \left(c_{t}\right)⊙o_{t} \end{array}$$

In the formula, *W* is the weight matrix, *b* is the offset, σ is the activation function (sigmoid), and φ is the activation function (tanh). The sigmoid function maps the output value of the model to the interval from zero to one, *x* represents the input value, and $F_{\mathrm{sgm}\;}\left(x\right)$ represents the output value. The output value will be judged as an abnormal label if it is closer to one. The calculation formula of the sigmoid function (fsgm) is as follows:

(4) $$F_{\mathrm{sgm}\;}\left(x\right)={\left(1+e^{-x}\right)}^{-1}$$

The decision tree is a common machine learning method. As the name implies, DT is based on a tree structure, which is a natural mechanism for human beings to use in dealing with decision problems [22]. In a decision tree, the root nodes are composed of samples, the intermediate nodes describe the decision process, and the leaf nodes are the final decision result. In this paper, the C4.5 decision tree algorithm is used to classify samples by gain ratio [23]. The formula for gain rate calculation is as follows:

(5) $$\begin{array}{c} Gain_{\mathrm{ratio}}\left(D,a\right)={\left(1+e^{-x}\right)}^{-1}\\ IV\left(a\right)=-\sum_{v=1}^{V}\frac{\left|D^{v}\right|}{\left|D\right|}\log_{2}\frac{\left|D^{v}\right|}{\left|D\right|} \end{array}$$

In the formula, $Gain_{\mathrm{ratio}}\left(D,a\right)$ represents the information gain obtained by partitioning sample collection *D* with attribute *a*. $D^{v}$ divides sample collection *D* with attribute *a*, and IV(a) is the intrinsic value of attribute *a*.

## 4. The Design of the LSTM-DT Hybrid Model

The LSTM-DT hybrid model is divided into three parts, which are introduced in detail below.

**Figure 2:**
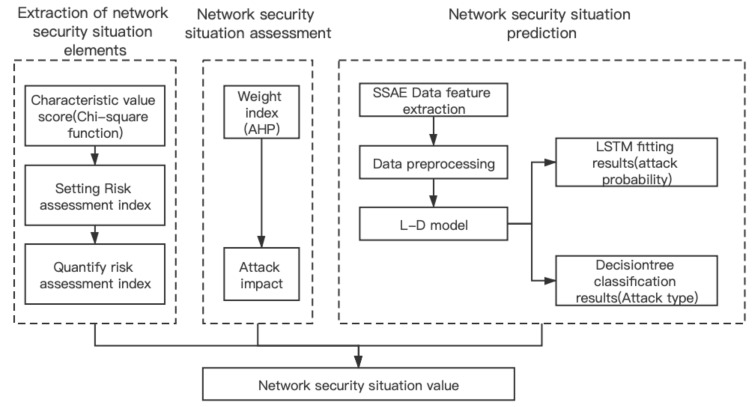
The design of the L-D network situation awareness model.

- Extraction of network security situation elements
  We evaluate the dataset by the chi-square function in order to set the network security situation elements and give the quantitative formula.
- Network security situation assessment
  The weight of the selected network security situation elements is calculated by the AHP method. At the same time, we give the attack influence degree of each attack mode.
- Network security situation prediction
  First, the dataset is extracted through the SSAE network to obtain new low-dimensional abstract features. Then the processed network traffic data are input into the LSTM-DT model in batches for training. The model is divided into two parts: fitting and classification. The LSTM network is used to make the fitting results. The prediction value is regarded as the probability of attack occurrence, and the tag value in the dataset is updated to serve the classification results, defining the type of network attack. Finally, the network security situation value is obtained by the product of attack probability and impact degree in the current time. The model is shown in Figure 2.

The time and space complexity of this model are mainly affected by the DT algorithm and the LSTM algorithm. The time complexity of the DT algorithm depends on the number of features, sample size, and tree depth. The number of multiplication and addition operations in the network affects the time complexity of the LSTM algorithm. In terms of space complexity, the DT algorithm is affected by sample size, feature number, and feature segmentation points. The LSTM algorithm is represented by the number of parameters to be optimized and layers in the neural network.

## 5. Extraction and Evaluation of Network Security Situation Elements

Referring to the network situation awareness index system constructed by others and combined with the common vulnerability scoring system (CVSS) [24], this paper proposes a quantitative method concerning the impact degree of attack based on the AHP as follows:

- Determine the risk assessment indicators.
- Construct the pairwise comparison matrix for the evaluation indexes and obtain their respective weights by the AHP.
- Calculate the risk assessment index for each attack based on the eigenvalues of training sets.
- Obtain the impact of each attack through the attack risk assessment index and its corresponding weight.

### 5.1. Extraction of Network Security Situation Elements

The chi-square function is used to score the eigenvalues of the UNSW-NB15 dataset. The several eigenvalues with the highest correlation coefficient between eigenvalues and attack types are the source sequence number (stcpb), target sequence number (dtcpb), source bits per second (sload), target bits per second (dload), flow rate (rate), target-to-source transaction bytes (dbytes), arrival time between source packets (sinpkt), and source-to-target transaction bytes (sbytes). Thus, we set the risk assessment indicators as packet loss rate, data traffic rate of change, and data throughput of the network. The weights of these three attribute values need to be calculated by AHP. Then, the influence degree of each attack is obtained through the multiattribute utility theory. The formula for the influence degree of each attack is as follows:

(6) $$F\left(j\right)=W_{L}{}^{*}U\left(L_{j}\right)+W_{R}{}^{*}U\left(R_{j}\right)+W_{T}{}^{*}U\left(T_{j}\right)$$

where $W_{L}$, $W_{R}$ and $W_{T}$ are the weights of the three attributes, and the sum is 1. *U* is the corresponding utility value, U(x)=C/X, *X* is the corresponding grade score of the attribute, and *C* is usually 1.

#### 5.1.1. Packet Loss Rate

Packet loss rate refers to the ratio at which the source address sends packets to a destination address that does not receive packets. The quantitative formula for setting the packet loss rate of the network is as follows:

(7) $$L_{j}=\frac{\sum_{i=1}^{n_{j}}\left(\frac{L1_{i}}{s_{i}}+\frac{L2_{i}}{D_{i}}\right)}{2n_{j}}$$

where $L_{j}$ is the packet loss rate of the j-th attack mode(ten types of the attack, *j* = 10), $n_{j}$ is the total number of such attacks, $L1_{i}$ is the number of packets lost from the source address to the destination address, and $S_{i}$ is the total number of packets sent from the source address to the destination address. In the same way, $L2_{i}$ and $D_{i}$ indicate that the destination address sends packets to the source address. Then, we divide the packet loss rate $L_{j}$ into four levels, as shown in Table 1.

#### 5.1.2. Data Traffic Rate of Change

Data traffic refers to the amount of data transmitted on the network, and the rate refers to the change of the data transmitted on the network within a certain continuous time. The formula for setting the data traffic rate of change is as follows:

(8) $$R_{j}=\frac{\sum_{i=1}^{n_{j}}\frac{a_{(\tau -1)i}}{a_{\tau i}}}{n_{j}}$$

where *R* represents the rate of the j-th attack mode, n is the total number of attacks, $Q_{Ti}$ and $Q_{(T-1)i}$ are the traffic values of the network in the current time period T and T-1 in the previous continuous time, which is divided into four levels, as shown in Table 2.

#### 5.1.3. Data Throughput of the Network

Throughput refers to the maximum rate that the device can accept without frame dropping. The test method is sent a number of frames at a certain rate, and the frames transmitted by the device to be tested are calculated. If the number of frames sent is equal to the number received, the transmission rate will be increased and retested; otherwise, the transmission rate will be reduced and retested until obtaining the final result. The quantitative formula is as follows:

(9) $$T_{j}=\frac{\sum_{i=1}^{n_{j}}\left(\frac{T_{s}i}{Hs_{i}}+\frac{Td_{i}}{Hd_{i}}\right)}{2n_{j}}$$

where $T_{j}$ is the throughput of the j-th attack mode, $Ts_{i}$ is the flow packet size sent by the source address, and $Hs_{i}$ is the time of arrival. Similarly, $Td_{i}$ and $Hd_{i}$ represent the size and time of sending packets from the destination address to the source address. We then divide the throughput into four levels, as shown in Table 3.

### 5.2. Network Security Situation Assessment

AHP is a decision analysis method of hierarchical weight proposed by Professor T.L. Saaty of the University of Pittsburgh in the early 1970s [25]. It uses the prior knowledge of evaluators to sort the importance of existing indicators and obtains the weights of various risk indicators combined with subjective judgment and objective methods.

#### 5.2.1. Paired Comparison Matrix

Assuming the given m-th evaluation indexes, experts need to construct a paired judgment matrix *D* (matrix size is m * m). *D* contains element $d_{j}^{i}$, which is used to represent the importance of index $X_{i}$ relative to indicator $X_{j}$. We usually use the method of a nine-grade scale to value it, as shown in Table 4.

#### 5.2.2. Consistency Checking

The consistency checking is conducted on the weight results to determine whether they meet the consistency requirements. The consistency checking needs to work out two indicators CI and RI. CI is the negative average of the eigenvalues from the comparison matrix except the maximum eigenvalue. The solution formula of the consistency index CI [26] is as follows:

(10) CI=(λmax−n)/(n−1)

The smaller the CI (close to zero) is, the more consistent the comparison matrix will be. The RI value refers to the average random consistency index of the comparison matrix. Its value of the comparison matrix about the order 1–9 is shown in Table 5.

Considering the values of CI and RI, the random consistency ratio CR is defined as follows:

(11) CR=CI/RI

If CR is less than 0.10, the comparison matrix satisfies the consistency requirement.

## 6. Network Security Situation Prediction Algorithm

**Figure 3:**
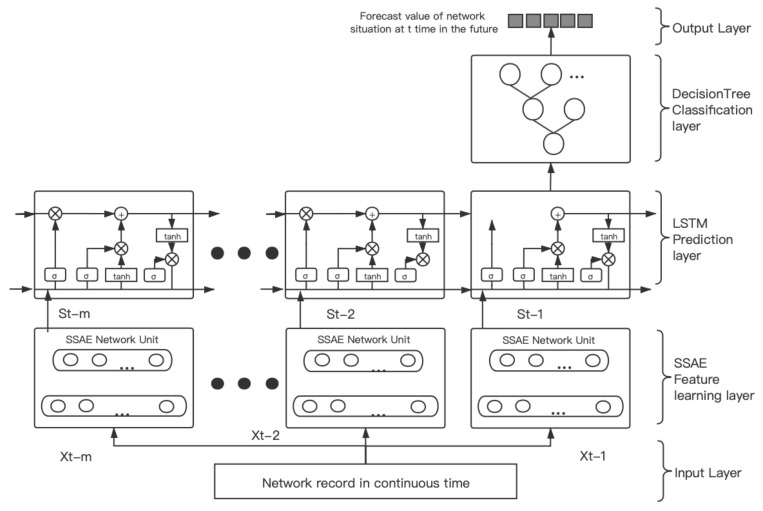
The structure of LSTM-DT network security situation awareness model.

The C4.5 decision tree algorithm is a very classic classification algorithm. Aiming at the timing of the network situation, we improve the decision tree algorithm presented in [27] and construct the LSTM-DT model algorithm of network situation awareness. The specific process is shown in Figure 3.

Given the network traffic dataset *D* = [$d_{1}$,$d_{2}$…$d_{n}$], take m-th data as the training sets, obtaining $D_{tr}$ = [$d_{1}$,$d_{2}$…$d_{m}$], the rest of the data are obtained as the testing sets $D_{te}$ = [$d_{m+1}$,$d_{m+2}$…$d_{n}$]. Based on SSAE, a new feature sequence ($S_{t-m}$,…,$S_{t-2}$,$S_{t-1}$) is obtained. Furthermore, the standardized training sets can be expressed as:

(12) $$\begin{array}{c} D_{tr}^{\prime }=\left\{s_{1},s_{2},\ldots ,s_{m}^{\prime }\right\}\\ d_{t}^{\prime }={\left(s_{t}-\sum_{t=1}^{n}s_{t}/n\right)}^{*}\\ \sqrt{\sum_{t=1}^{n}{\left(s_{t}-\sum_{t=1}^{n}s_{t}/n\right)}^{2}/n} \end{array}$$

LSTM ${}_{\mathrm{cell}}$ is created by cell state S ${}_{\mathrm{state}}$, and then LSTM ${}_{\mathrm{net}}$ is constructed. LSTM ${}_{\mathrm{net}}$ is initialized by seed. So far, the construction of the LSTM network has been completed. Nest input data are produced by batch processing, using the LSTM forward calculation method to obtain the predicted value. The LSTM network is updated by the loss rate and learning rate η, obtaining the trained network. In the prediction process, the iterative method is used to output the prediction value $P_{o}$ one by one, and the Z-score standardization is performed on $P_{te}$ to obtain the prediction sequence corresponding to the test set, which is the probability of attack occurrence. Use $P_{te}$ to update $D_{te}$ and generate a new test set $D_{te}^{\prime }$. $D_{tr}$ and $D_{te}^{\prime }$ are used as the input of the classification algorithm based on the DT algorithm, and the output results are ten attack types. Through the information gain calculation, we can obtain the attribute value $a_{best}$ of each tuple in the dataset, and the mapping relationship $D_{v}$ between the attribute value and category is obtained to complete the construction of the tree. The specific algorithm (Algorithm 1) is as follows.
**Algorithm 1** Network security situation awareness algorithm based on LSTM-DT model.Input: D, m,$S_{state}$, seed, steps, η, attribute *A*
Output: Prediction value, loss value and sort results corresponding to test set
1. get $D_{tr}$, $D_{te}$ from D by m
2. $S_{t}$ = SSAE ($D_{tr}$)
3. $D_{tr}$’ = zscore ($S_{t-m}$)
4. create $LSTM_{cell}$ by $S_{state}$
5. connect $LSTM_{net}$ by $LSTM_{cell}$
6. initialize $LSTM_{net}$ by seed
7. for each step in 1:steps
8. P = $LSTM_{forward}$($D_{tr}$’)
9. $\mathrm{Loss}=\sum_{i=1}^{m}{\left(p_{i}-y_{i}\right)}^{2}/m^{2}$
10. update $LSTM_{net}$ by Loss and η
11. get $LSTM_{net}$*
12. for each j in 0:(n − m − 1)
$P_{f+i}$ = $LSTM_{net}$*(P) append $P_{O}$ with $P_{f+i}$[−1]
13. $P_{te}$ = de_zscore ($P_{O}$)
14. update $D_{te}$ by $P_{te}$
15. get $D_{te}$’
16. Tree = Create root node *n*
17. for all attribute *A*∈$D_{te}$’ do
Use compute information-theoretic criteria
get $a_{best}$
18. end for
19. Tree = Create node that tests $a_{best}$ in the root
20. get $D_{v}$ from $D_{te}$’ based on $a_{best}$
21. for all $D_{v}$ do
$Tree_{v}$ = C4.5($D_{v}$)
Attach $Tree_{v}$ to the corresponding branch of Tree
22. end for
23. return sort results(attack type)

## 7. Datasets and Experiments

### 7.1. Data Sources

**Figure 4:**
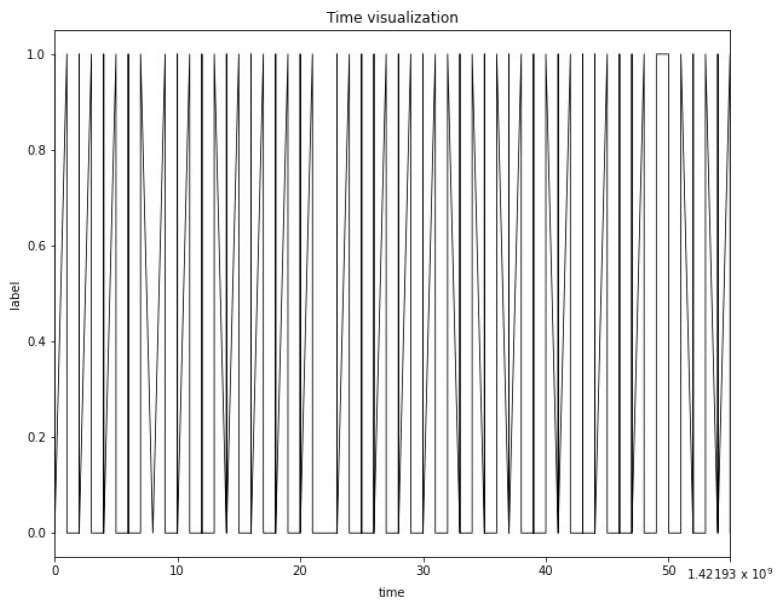
Time visualization image of the UNSW-NB15 dataset.

This paper uses the UNSW-NB15 public dataset, which is created by the Ixia PerfectStorm tool of the network-wide laboratory and is used to generate a mixture of real modern normal activities and synthetic contemporary attack behaviors. The Tcpdump tool is used to capture 100 GB of raw traffic (such as a PCAP file). The dataset has nine types of attacks, which are Fuzzers, Analysis, Backdoors, Dos, Exploits, Generic, Reconnaissance, Shellcode, and Worms. Using Argus and bro IDS tools, 12 algorithms are developed to generate 49 features with class tags. This dataset has a time feature (stime), and it is continuously distributed in the time dimension. Therefore, choosing to build the LSTM time series mode as a prediction method can reflect its unique advantages. Figure 4 shows the image of time visualization for the dataset. It can be seen from Figure 4 that the network security situation fluctuates back and forth between 0 to 1 in the continuous time.

### 7.2. Extraction of Network Situation Elements and Analysis of Evaluation Results

#### 7.2.1. Extraction Results of Network Situation Elements

In this paper, the network situation elements are extracted as network data packet loss rate, data traffic rate of change, and data throughput of the network. According to their respective quantitative formulas, the extraction results are shown in the following table. According to Equation (7) and Table 1, the packet loss rate and corresponding level of various attacks can be obtained, as shown in Table 6.

According to Formula (8) and Table 2, the average rate and corresponding level of various attacks can be obtained, as shown in Table 7.

According to Formula (9) and Table 3, the throughput and corresponding level of various attacks can be obtained, as shown in Table 8.

#### 7.2.2. Network Situation Assessment Results

By using the nine-scale method and referring to the results of the chi-square test, the pairwise comparison matrix A = [$\begin{array}{ccc} 1 & 3 & 2\\ 1/3 & 1 & 1/2\\ 1/2 & 2 & 1 \end{array}$] of three types of risk indicators is constructed through expert scoring, and then the weights are obtained by the AHP algorithm as $W_{L}$ = 0.54,$W_{R}$ = 0.16, and $W_{T}$ = 0.30. The consistency test showed that CI = 0.0046, CR = 0.0079, which met the requirements of consistency. The threat attribute scores of 10 types of attacks are given respectively. Thus, the attack impact degree F of each type of attack is calculated according to Formula (6) as shown in Table 9.

### 7.3. Analysis of Network Situation Prediction Results

#### Network Attack Probability Analysis

**Figure 5:**
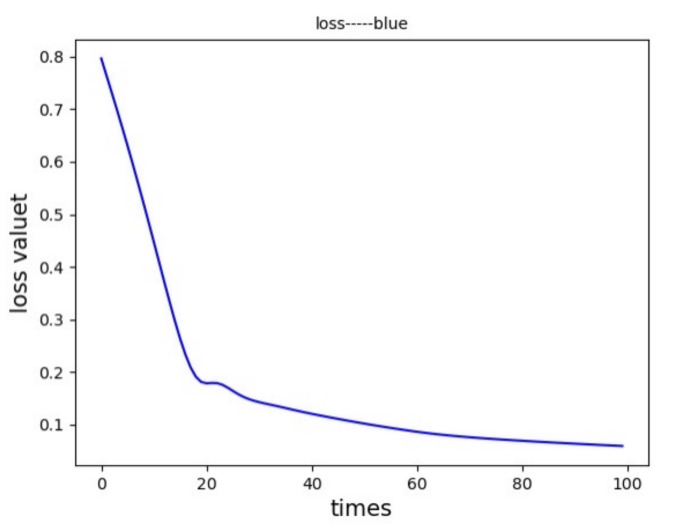
Model training effect.

The probability of network attack is mainly predicted by the LSTM time series model. In the training process of the dataset, the loss function is used to measure the difference between the estimated value and the observed value of the model. The formula is as follows:

(13) $$\mathrm{loss}\;:\;L(\hat{y},y)=\omega \left(\theta \right){\left(\hat{y}-y\right)}^{2}$$

where ω(Θ) is the weight of the real value, *y* is the real value, and $\hat{y}$ is the output of the model. Figure 5 shows the image of the loss function when training the dataset after applying the LSTM model. It can be seen that the development trend of the loss function is zero, and the training effect is good

**Figure 6:**
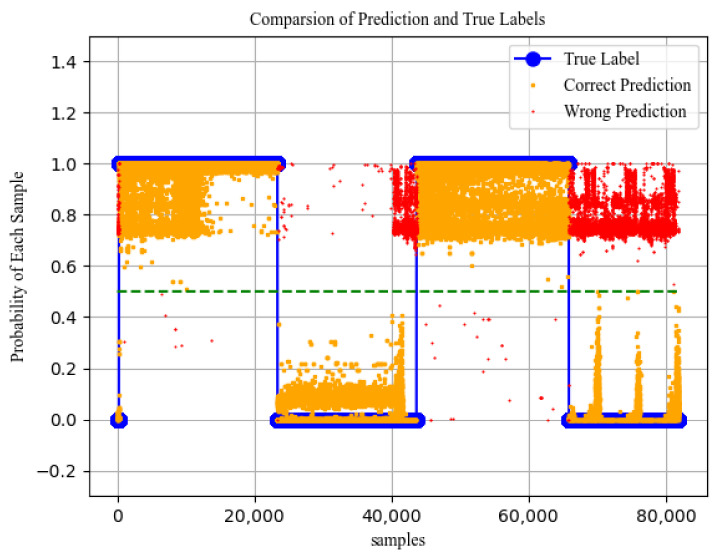
LSTM model fitting image.

Testing the prediction effect of the LSTM-DT model through the early training, we can obtain the predicted value and fitting effect, as shown in Figure 6.

**Figure 7:**
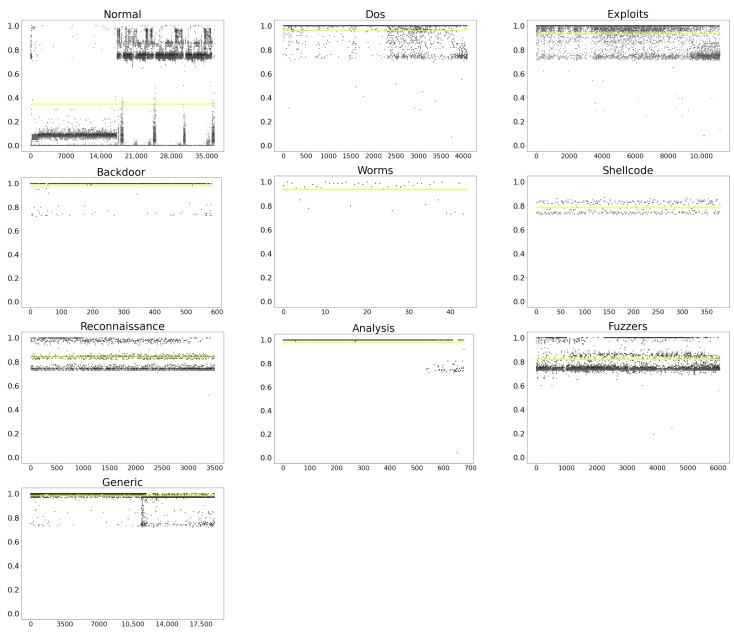
Attack probability of various attacks.

It can be seen from Figure 7 that the closer the threat predicted value is to 1, the greater the possibility of attack is. In addition, the threshold of attack probability is conventionally defined as 0.5, so if the attack probability is over 0.5, we can see the label as 1. Otherwise, the label is 0. According to the prediction process of the dataset, the accuracy is used to evaluate the fitting results, and the final prediction accuracy is 87%. The predicted value of the model is regarded as the probability of threat occurrence. This improvement makes the threat classification no longer a simple yes or no but has a certain probability flexibility. The higher the probability of occurrence is, the more serious the threat will be. The probability of threat occurrence is defined as follows:

(14) $$P{\left(a\right)}_{t}=\left\{\begin{array}{cc} P(a), & \mathrm{vlus}{}_{j}\in \;\mathrm{Vlus}\;\\ 0, & \mathrm{Others} \end{array}\right.$$

where $vlus_{j}$ is the specific attack mode, and Vlus is all attack modes existing in the network. Figure 7 shows the probability of each attack, and the scatter diagram is composed of the predicted value. The yellow solid line represents the average probability of each attack.

From the scatter number in Figure 7, we can see that the data amount of Normal is the largest, and the Worms is the smallest. According to the concentration of scattered points, Backdoor and Analysis can be compared to predict better. By comparing the position of the yellow solid line in the graph, it can be concluded that the attack probability of Generic is the highest, which is 0.984; the attack probability of Normal is the lowest, which is 0.345.

## 8. Analysis and Comparison

### 8.1. Analysis and Comparison of Classification Results

Previous scholars used the random forest and neural network to identify attack types. We apply this to UNSW-NB15 dataset, and the results show that the multiclassification accuracy was 74%. In this paper, the SSAE network is used for feature learning, and the DT model is used for multiclassification, which not only reduces the cost of the model but also improves the classification accuracy to 87%.

**Figure 8:**
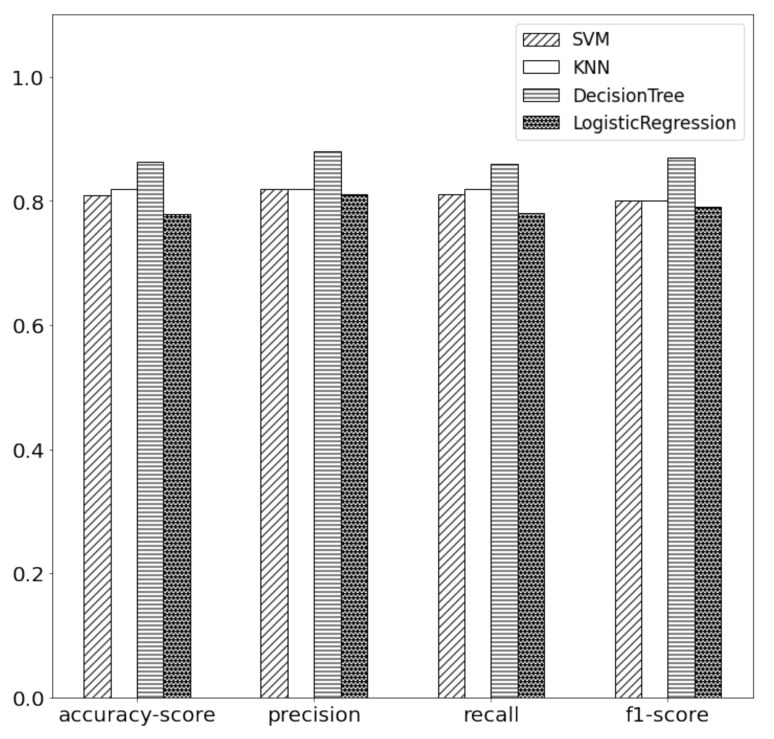
Classification result evaluation of each classifier.

To study the multiclass classification effect of classifier categories on network attack types, experiments are carried out in four different classifiers, which are SVM, k-nearest neighbor(KNN), DT, and logistic regression (LR). Here we only use the basic structure of each model for experiments, without using the improved model structure. Future work can increase the types of comparison or change the existing model structure to achieve better results. Then, the accuracy score, precision, recall, and F1 score are used as the evaluation indexes for the experimental results. From Figure 8, we can see that the DT has a good effect on the network threat test, so we choose the decision tree as a multiclass classification tool.

**Figure 9:**
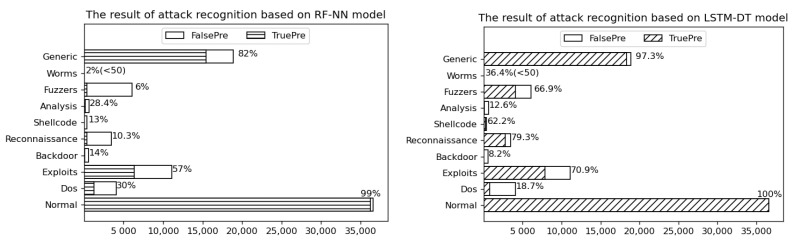
Accuracy of identifying various attacks.

The classification accuracy of each attack is shown in Figure 9.

As can be seen from Figure 9, the RF-NN method depends on the size of the data sample. For the type of attack with a small size, the recognition accuracy will decrease significantly, so the model stability is poor. Although the model method proposed in this paper is also affected by the number of samples, the accuracy of Worms (minimum number of samples) is still 36.4%, which is much better than the former. Normal has the highest classification accuracy and it accounts for the largest proportion in the testing sets. Secondly, the types of attack with high accuracy are Generic, Reconnaissance, and Exploits, and the accuracy is 97.3%, 79.3%, and 70.9%. Some attacks such as Worms only have 44 records in the testing sets and 174 records in the training sets. Due to the lack of samples, the classification result is relatively poor. Thus, the more records each attack has, the higher the accuracy will be.

### 8.2. Analysis and Comparison of Network Security Situation Awareness

The network security situation value (NSSV) is calculated by the attack influence degree F and the attack possibility P. It is defined as follows:

(15) $$N_{t}=\frac{\sum_{a=1}^{n}N_{ta}}{n}=\frac{\sum_{t=1}^{n}F_{ta}*P\left(a\right)t}{n}$$

$N_{t}$ represents the network security situation value in a certain of continuous time, and *a* represents the number of specific records contained in each situation, here regarded as the window value. $P{\left(a\right)}_{t}$ represents the attack probability, and $F_{ta}$ is the attack influence degree corresponding to the attack type identified at this time. The calculation results are shown in Table 10.

**Figure 10:**
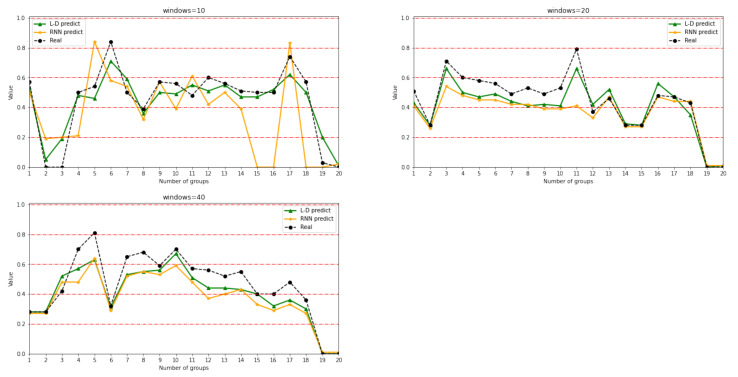
20 groups of network security situation values under different window sizes.

The values of *a* in Formula (15) are 10, 20, and 40, respectively, meaning that every 10, 20, and 40 in a continuous time is a group. Additionally, we extract 20 network situation values from each group to obtain the network security situation and draw a line chart, so as to see the overall security trend of the network. Referring to the *National Emergency Plan for Public Emergencies*, the network security situation value is divided into five intervals (0.00–0.20, 0.21–0.40, 0.41–0.60, 0.61–0.80, and 0.81–1.00), and the severity degree is security, low risk, medium risk, high risk, and super risk. The result can be seen in Figure 10.

Figure 10 shows that under three different window values, the network security situation values calculated by the LSTM-DT model are more close to the actual operation of the network. Owing to the LSTM being improved by the RNN network, the network security situation awareness method proposed in this paper is applied to the basic RNN network, and the obtained situation value is compared with the LSTM-DT model. Then, we calculate the network security situation values and draw charts compared with the LSTM-DT model.

Taking a window equal to 10 as an example, although the results of the LSTM-DT model are slightly different from the real value in 20 groups, 19 groups of data fall within the same range of the network security situation compared with the real value, obtaining an accuracy rate of 95%. The accuracy rate of the original RNN model is only 80%. This is because the LSTM-DT model applies SSAE feature learning, which makes the efficiency of data prediction higher. On the other hand, the combination of DT and LSTM makes the accuracy higher. Table 11 compares the accuracy, precision, recall, and F1 values of the L-D model and RNN model under different window values.

## 9. Conclusions

Experiments show that the proposed network security situation awareness method not only can identify the specific attacks faced by the current network but also can quantify the impact of attacks. The results of situational awareness are closer to the actual operation of the network. The NSSA algorithm based on the LSTM-DT model is feasible, and the next step can improve the accuracy of the algorithm by comparing it with various classic algorithms. For example, the super parameter optimization method is introduced to set the cell state vector size and learning rate of the LSTM network more scientifically.

In this paper, the determination of the threshold value of network attack probability is also worth discussing. We regard the threshold value as 0.5 according to popular agreement. The next step is to increase the experimental amount and find the threshold that makes the fitting accuracy reach more than 90%. Moreover, due to the uneven distribution of attack types in the network traffic data, the multiclass classification problem with unbalanced data can be considered to optimize the experimental results.

## Figures and Tables

**Table 1:** Risk indicators of the loss.

Risk Indicators	Risk Level
Loss > 0.002	The first rank
Loss > 0.001	The second rank
Loss > 0	The third rank
Loss = 0	The fourth rank

**Table 2:** Risk indicators of the rate.

Risk Indicators	Risk Level
Rate (<10000)	The first rank
Rate (10,000–50,000)	The second rank
Rate (50,000–100,000)	The third rank
Rate (>100,000)	The fourth rank

**Table 3:** Risk indicators of the throughput.

Risk Indicators	Risk Level
Throughput (<500)	The first rank
Throughput (500–5000)	The second rank
Throughput (5000–10,000)	The third rank
Throughput (>10,000)	The fourth rank

**Table 4:** Nine-grade scale method.

Scale	Meaning
1	The two indicators have the same importance
3	The former is slightly more important than the latter
5	The former is a bit more important than the latter
7	The former is more important than the latter
9	The former is much more important than the latter
2,4,6,8	The median value of the above adjacent judgment
reciprocal	If $X_{i}$ is compared with $X_{j}$ to obtain $d_{j}^{i}$, then $X_{j}$ is compared with $X_{i}$ to obtain 1/$d_{j}^{i}$

**Table 5:** RI value of comparison matrix of order 1–9.

1	2	3	4	5	6	7	8	9
0.00	0.00	0.58	0.90	1.12	1.24	1.32	1.41	1.45

**Table 6:** Each attack type corresponds to the packet loss rate level.

NORM	Generic	RCN	Exploits	Fuzzers	Dos	ANLS	Worms	Backdoors	Shellcode
0	0.00057	0.00184	0.00102	0.00319	0.00084	0	0.00187	0.00125	0.00206
4	3	2	2	1	3	4	2	2	1

**Table 7:** Each attack type corresponds to the average rate level.

NORM	Generic	RCN	Exploits	Fuzzers	Dos	ANLS	Worms	Backdoors	Shellcode
121,829	195,836	43,363	50,718	39	14,305	137,064	36,705	55,580	45,423
4	4	2	3	1	2	4	2	3	2

**Table 8:** Each attack type corresponds to the throughput level.

NORM	Generic	RCN	Exploits	Fuzzers	Dos	ANLS	Worms	Backdoors	Shellcode
36,974	5581	1821	1896	2	487	6753	9008	2656	3238
4	4	2	2	1	1	3	3	2	2

**Table 9:** Attack impact of each attack type.

Attack Mode	Loss	Rate	Throughput	Attack Impact
Normal	4	4	4	0.25
Generic	3	4	4	0.30
Reconnaissance	2	2	2	0.5
Exploits	2	3	2	0.473
Fuzzers	1	1	1	1
Dos	3	2	1	0.56
Analysis	4	4	3	0.275
Worms	2	2	3	0.45
Backdoors	2	3	2	0.473
Shellcode	1	2	2	0.77

**Table 10:** Network security situation values 715–725.

Attack Probability	Attack Type	Attack Impact	Network Security Situation Value
1.00	Exploits	0.473333333	0.473
1.00	Exploits	0.473333333	0.473
1.00	Dos	0.56	0.560
1.00	Exploits	0.473333333	0.473
0.97	Exploits	0.473333333	0.459
0.96	Exploits	0.473333333	0.454
1.00	Exploits	0.473333333	0.473
1.00	Exploits	0.473333333	0.473
1.00	Fuzzers	1	0.740
0.74	Exploits	0.473333333	0.473

**Table 11:** Comparison between L-D model and RNN model.

Windows	Model	Accuracy	Precision	Recall	F1
10	L-D	0.95	1	0.95	0.97
	RNN	0.80	0.73	0.80	0.76
20	L-D	0.90	0.90	0.90	0.90
	RNN	0.65	0.72	0.65	0.61
40	L-D	0.80	0.84	0.80	0.78
	RNN	0.50	0.67	0.50	0.51

## Data Availability

Not applicable.
